# Case report: Thrombotic thrombocytopenic purpura in a pregnant woman with lupus membranous nephropathy: a diagnostic challenge

**DOI:** 10.3389/fneph.2024.1343594

**Published:** 2024-02-05

**Authors:** Marina Leiva, Gustavo Navarro, J Daniel Carpio, Leopoldo Ardiles

**Affiliations:** ^1^ Department of Nephrology, Hospital Intercultural de Nueva Imperial, Nueva Imperial, Chile; ^2^ Laboratory of Nephrology, Institute of Medicine, Faculty of Medicine, Universidad Austral de Chile, Valdivia, Chile; ^3^ Institute of Anatomy, Histology and Pathology, Faculty of Medicine, Universidad Austral de Chile, Valdivia, Chile

**Keywords:** thrombocytopenia, microangiopathic anemia, lupus (SLE), acute kidney injury, ADAMST 13

## Abstract

A 27-year-old female at 20th week of pregnancy was admitted with edema, foamy urine, but normal blood pressure. Her blood count was normal, she had proteinuria of 3 g/day, creatinine 0.4 mg/dl, albumin 2.4 g/dl, and cholesterol 355 mg/dl. Antinuclear antibodies 1/160, but Anti-DNA, anticardiolipin antibodies and lupus anticoagulant were negative, with normal serum C3 and C4. A renal biopsy showed secondary membranous glomerulopathy, most likely lupus class V pure. Steroids, azathioprine, and aspirin were initiated, up to 28 weeks of pregnancy, when she developed severe hypertension, photopsia, headache, anasarca, extensive bruising of the extremities, severe anemia, thrombocytopenia, and creatinine rose to 2.09 mg/dl with preserved diuresis. A female infant, 1045 grams, was delivered by emergency caesarean section. Following the surgery, she experienced diplopia, dysarthria, bradypsychia, and sensory alterations in the lower extremities, necessitating emergency hemodialysis due to pulmonary congestion. Blood smear revealed schistocytes, LDH elevated at 1148 IU/L, while transaminases and liver function remained normal, suggesting thrombotic thrombocytopenic purpura. ADAMTS13 revealed 6% activity with the presence of inhibitor. Mycophenolate and daily plasmapheresis with fresh frozen plasma replacement yielded unsatisfactory response, unaffected by the addition of methylprednisolone pulses and rituximab. Eventually, intravenous cyclophosphamide was introduced, resulting in complete hematological remission and normalization of ADAMTS13, however dialysis-dependence persisted and four years later, right renal cancer prompted bilateral nephrectomy. After a total follow-up of six years, she remained free of neoplastic recurrence and lupus activity, receiving prednisone and hydroxychloroquine. The differential diagnosis of microangiopathic syndrome in a pregnant lupus patient is discussed.

## Introduction

Thrombotic microangiopathy (TMA) is a potentially serious condition characterized by endothelial cell injury. Diagnosis is primarily based on clinical and biological data, which typically involve a classic triad of peripheral thrombocytopenia, mechanical hemolytic anemia, and organ dysfunction, especially in the central nervous system (e.g., altered consciousness, seizures), the kidneys (e.g., acute kidney injury), and the heart (1). TMA is identified by detecting a low platelet count, decreased hemoglobin levels, raised lactate dehydrogenase (LDH) serum levels, undetectable serum haptoglobin, negative direct erythrocyte antiglobulin test, schistocytes in blood smears, or the demonstration of TMA characteristics in kidney or other organ biopsies (1).

Pregnancy and postpartum are high-risk periods for various forms of thrombotic microangiopathy (TMA) with a range of causative factors. These can be classified into two categories: those exclusively observed during pregnancy (e.g. preeclampsia) and those that can also occur in nonpregnant individuals, but pregnancy acting as a trigger. Although clinical overlap exists, management strategies differ considerably (2).

The primary causes of TMA in pregnancy are preeclampsia (PE)/eclampsia and hemolysis, elevated liver enzymes, and low platelets (HELLP) syndrome. These conditions are interrelated but differ in presentation and severity. Acute fatty liver disease, hemolytic uremic syndrome (HUS; a condition where the kidneys are predominantly affected), and thrombotic thrombocytopenic purpura (TTP; with predominant hematological and neurological involvement) are less common. It can be present in severe autoimmune diseases, especially systemic lupus erythematosus (SLE) and catastrophic antiphospholipid syndrome (CAPS) (1).

The approach to pregnancy-related thrombotic microangiopathy (TMA) includes two main objectives. The first is to conduct an immediate ADAMTS13 activity test to confirm or rule out TTP, due to its potential life-threatening risks. The second is the prompt diagnosis of complement-mediated atypical hemolytic uremic syndrome (aHUS) in order to initiate specific treatment (1).

TMA in pregnancy constitutes a medical emergency, with delays in diagnosis and treatment resulting in potentially life-threatening consequences. Maternal complications encompass a range of adverse outcomes such as renal failure, seizure, stroke, pulmonary edema, disseminated intravascular coagulation (DIC), requiring blood transfusion, ICU admission, and mortality. Timely decision-making could be hindered due to the presence of overlapping clinical features and diagnoses across multiple disciplines, such as Hematology, Nephrology, and Maternal Fetal Medicine. Given the causal variability and the incomplete clarity of the specific pathogenic mechanisms in each of the clinical entities responsible for TMA in pregnancy, diagnosing and treating the condition is still challenging (3).

## Case report

**Figure 1:**
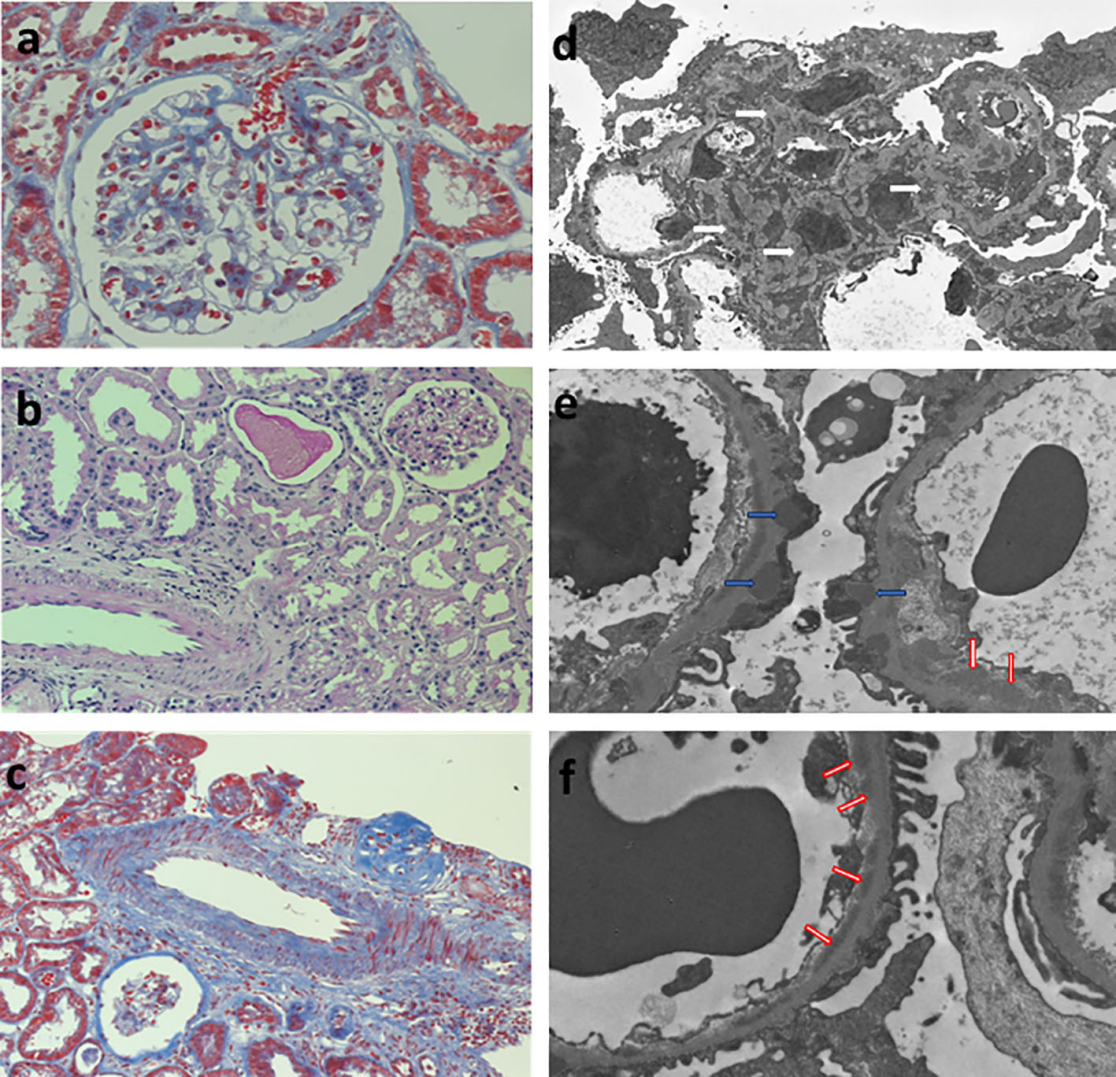
Renal biopsy. Left column, light microscopy: **(A)** glomerulus showing mild thickenning of the capillary walls and mesangial hypercelularity (Periodic Acid-Schiff Stain 400x), **(B)** normal tubulointerstitial compartment (Periodic Acid-Schiff Stain 200x), **(C)** normal médium size artery (Masson´s trichromic stain 200x). Right column, glomerular inmune deposits in **(D)** mesangial (white arrows), **(E)** subepithelial (blue arrows), and **(F)** subendothelial (red arrows) localization.

**Figure 2:**
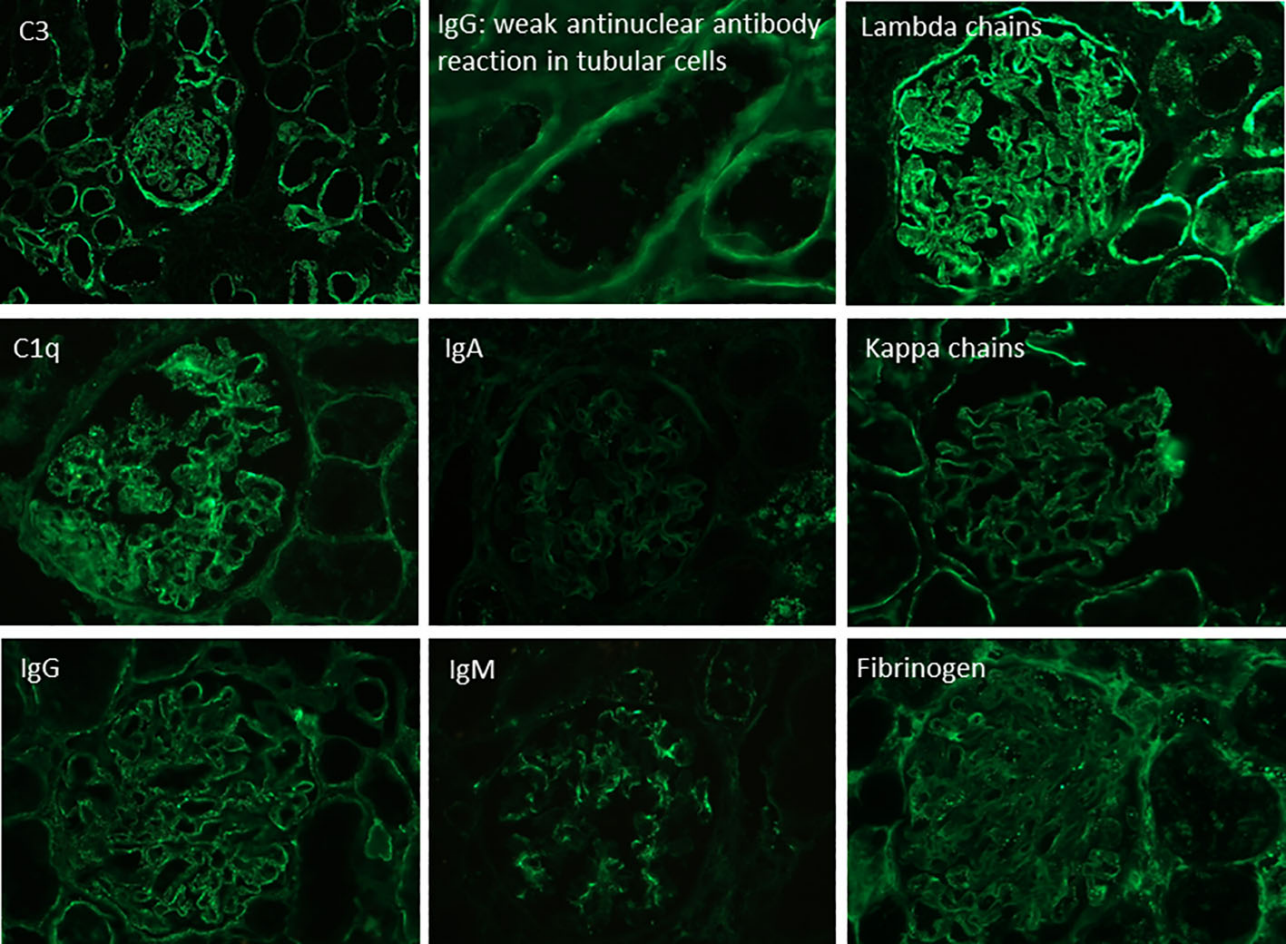
Direct immunofluorescence in frozen renal tissue showing a “full house” pattern.

**Figure 3:**
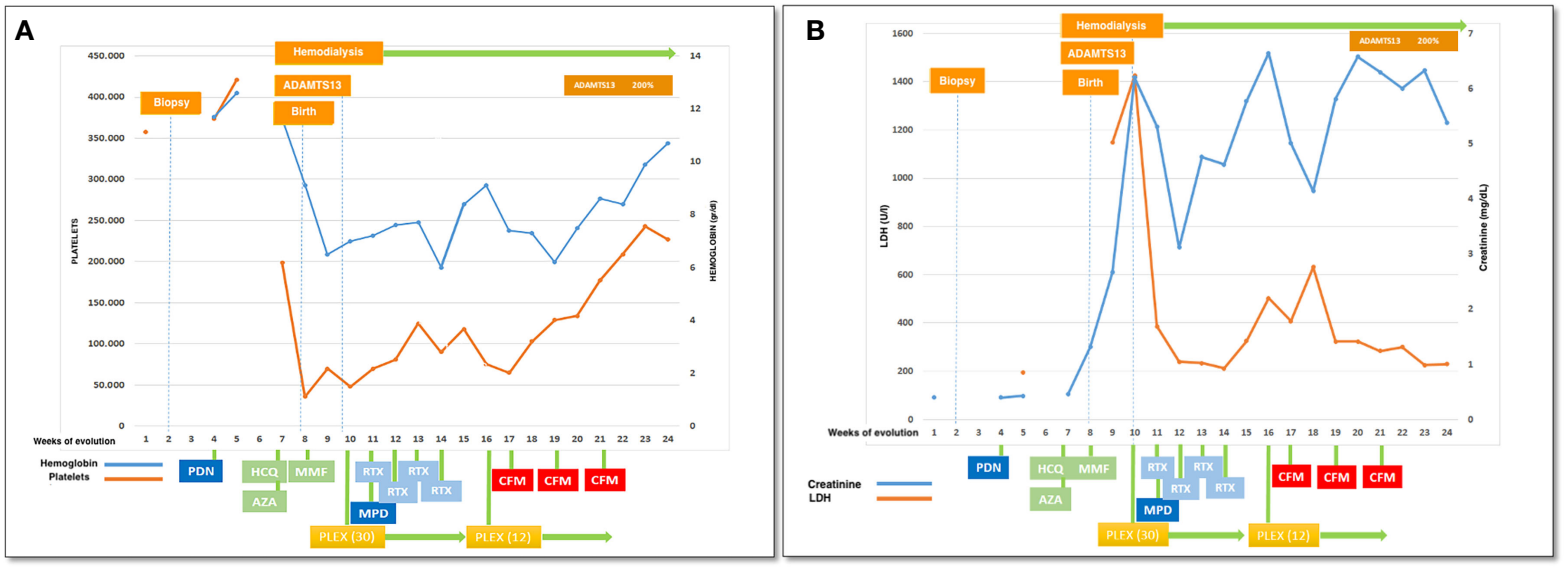
Clinical evolution: **(A)** platelets and hemoglobin, **(B)** LDH and creatinine in relationship with therapeutical interventions. PDN, prednisone; HCQ, hydroxychloroquine; AZA, azathioprine; MMF, mycophenolate mophetil; PLEX, plasma exchange (number of sesiones); RTX, rituximab; CFM, cyclophosphamide iv pulses.

A 27-year-old woman, previously in good health and 20 weeks into her pregnancy, had normal blood pressure and routine check-ups during her prenatal care at primary health facilities. However, one week prior to hospitalization, she was showing symptoms of edema in her extremities and foamy urine. Upon admission, the patient exhibited a blood pressure of 124/68 mmHg, a heart rate of 80 bpm, and a fetal heartbeat of 140 bpm, alongside severe edema of the lower extremities. The laboratory tests indicated normal blood count, proteinuria of 3 g/24 hours, creatinine of 0.4 mg/dl, uric acid 4.3 mg/dl, albumin of 2.4 g/dl, and total cholesterol of 355 mg/dl. The antinuclear antibodies were positive at a rate of 1/160, while anti-DNA, C3, and C4 complement factors were normal, and the IgG and IgM anticardiolipin antibodies and lupus anticoagulant were negative. A pure nephrotic syndrome was diagnosed, antibodies to the phospholipase A2 receptor (PLA2R) resulted negative and a renal biopsy was performed revealing 1 out of 4 globally sclerotic glomeruli with thickened membranes, mesangial proliferation, protein reabsorption drops in tubules, scant lymphocytic infiltrate, tubular atrophy, and discrete interstitial fibrosis (**Figure 1**). Immunofluorescence demonstrated a full-house pattern positivity for IgG, IgM, IgA, C3, C1q, fibrinogen, kappa, and lambda light chains, in addition to an IgG weak antinuclear antibody-type extraglomerular reaction (**Figure 2**). Electron microscopy confirmed the existence of significant, continuous subepithelial, mesangial and subendothelial electrodense deposits also (**Figure 1**), which were associated with an increase in mesangial matrix and cells. These findings suggested that the patient was experiencing a secondary membranous glomerulopathy, possibly caused by lupus, specifically pure class V; therefore, corticosteroid therapy, azathioprine and hydroxychloroquine, combined with aspirin, were initiated. During the 28th week of pregnancy, she experienced severe hypertension, photopsia and headache, anasarca, extensive bruising of the extremities, severe anemia, and thrombocytopenia. These symptoms were accompanied by a rise in serum creatinine up to 2.09 mg/dl, and elevation of uric acid to 8.0 mg/dl, although diuresis was preserved. Suspecting severe preeclampsia, an infusion of magnesium sulfate was initiated, and an emergency caesarean section was performed, resulting in the birth of a female infant weighing 1045 grams, appropriate for gestational age. During the post-surgery period, the patient exhibited diplopia, dysarthria, bradypsychia, and sensory changes in their lower limbs, with renal impairment necessitating urgent hemodialysis due to pulmonary congestion. The complete blood count revealed schistocytes and an LDH value of 1148 IU/L, with normal transaminases as well as preserved liver function and coagulation tests, normalizing uric acid levels to 4.1 mg/dl. The suspected diagnosis was thrombotic thrombocytopenic purpura (TTP), for which a functional test was conducted to determine the activity of A Disintegrin And Metalloprotease with ThromboSpondin type 1 repeats 13 (ADAMTS13) that showed only 6% activity (normal values ranging from 41-180%) with the presence of an inhibitor (titer not determined). Sodium mycophenolate at a dose of 1 g every 12 hours was initiated, and daily plasmapheresis was conducted with replacement of fresh frozen plasma. However, due to an inadequate response, pulses of methylprednisolone and Rituximab (375 mg/m2) were commenced every week for four weeks while maintaining daily plasmapheresis until 30 sessions were completed. Nonetheless, the patient continued to suffer from severe anemia and thrombocytopenia. Therefore, the treatment was altered to include intravenous cyclophosphamide (three doses of one gram), resulting in complete hematological remission, normalization of anemia, stable platelet counts, and a return to normal ADAMST13 activity (200%) with disappearance of the inhibitor; however, dialysis-dependent renal failure remained persistent (**Figure 3**).

Four years later, while on stable hemodialysis and undergoing evaluation for kidney transplant candidacy, an ultrasound of the abdomen revealed a mixed solid and cystic tumor measuring 96 x 85 mm in the right kidney. This finding was confirmed by contrast-enhanced computed tomography, which further characterized the lesion as nodular with central necrosis, as well as demonstrating an atrophic appearance and multiple cystic formations in the left kidney. A successful bilateral laparoscopic radical nephrectomy was carried out without any complications, revealing the presence of clear-cell renal carcinoma. In the left kidney, where the renal biopsy was conducted, the findings were indicative of chronic sclerosing nephritis with fibro-cellular crescents and chronic tubulointerstitial involvement, along with moderate to severe arteriosclerosis and arteriolosclerosis. After six years of follow-up, the patient remains on hemodialysis, and there is no evidence of neoplastic disease or lupus activity recurrence; the patient is taking 2.5 mg prednisone and 200 mg hydroxychloroquine.

## Discussion

This case exemplifies the challenges in distinguishing a microangiopathic syndrome in a pregnant, formerly healthy young woman, who had recently been diagnosed with lupus, initially affecting only the kidneys not distressing the pregnancy itself, as evidenced by an appropriate fetal growth.

The presence of mesangial proliferation observed in optic microscopy, the full house pattern and antinuclear staining in tubular cells at direct immunofluorescence, and the presence of immune deposits in electron microscopy, including large continuous subepithelial, mesangial, and subendothelial deposits, were suggestive of a secondary etiology for the renal lesion (4). Antinuclear antibodies at an abnormal level, even with negative anti-DNA antibodies and normal serum complement C3 and C4 levels, is a common finding in pure membranous (class V pure) lupus nephropathy (4). While no specific antigen (5) was identified as the cause of the glomerular lesion, the clinical presentation, histological features, full-house pattern of immunofluorescence, ultrastructural findings, and subsequent progression strongly support the lupus etiology.

By the 28th week of pregnancy, severe hypertension and neurological symptoms appeared, along with evidence of a microangiopathic syndrome. The most likely diagnosis, based on frequency, was the most severe form of eclampsia, the HELLP syndrome (6), however, our patient’s liver enzymes were normal, and the onset of symptoms was somewhat early.

The normality of liver tests, including the absence of jaundice, normal levels of fibrinogen and prothrombin, and the early onset of anemia and thrombocytopenia, suggested that the possibility of an acute fatty liver was unlikely (6, 7). Systemic viral infections, such as disseminated herpes simplex, are exceedingly rare during pregnancy and typically presents during the 3rd trimester without signs of hypertension and is primarily associated with liver-related symptoms (8). VIH (9) was not detected, and the hemodynamic profile was not consistent with septic shock either (10). Given the presence of lupus, the potential for secondary catastrophic antiphospholipid syndrome (11) could not be ignored; however, both anticardiolipin antibodies and lupus anticoagulant factor were found to be absent. The range of thrombotic microangiopathies as a complication of lupus is wide, spanning from laboratory changes without clinical significance or incidental findings in renal biopsies, to antiphospholipid syndrome or the severe condition of immune-acquired TTP (iTTP) with reduced ADAMST13 activity (12). Indeed, in lupus it is possible to demonstrate antibodies against ADAMTS13 that cannot reduce its activity suggesting that the effectiveness of the antibody and the molecule domain it targets may play a role (13). On the other hand, renal microangiopathic complications are not frequent in pure membranous lupus nephropathy (14). Taking into account all the above, iTTP is life-threatening and requires a combined therapy, including plasmapheresis to remove the antibody and intensive immunosuppression to reduce its production.

The only medication utilized by the patient that may be associated with thrombotic microangiopathy (TMA) was hydroxychloroquine (15), a derivative of quinine, that has not caused a reoccurrence of the phenomenon, even with ongoing use to date.

Thrombotic thrombocytopenic purpura (TTP) and hemolytic uremic syndrome (HUS) are highly uncommon microangiopathic disorders during pregnancy and postpartum (< 1/100000 pregnancies) and, typically, these conditions are reported as either case reports or small case series (6). The pathophysiological basis of these syndromes entails platelet aggregation in the arteriolar and capillary walls, coupled with endothelial injury. Pregnancy-associated hemolytic uremic syndrome (HUS) is a subtype of atypical HUS that manifests more commonly during the postpartum period (16). TTP is a rare condition linked to either an inherited or acquired deficit of ADAMST13, a molecule involved in the cleavage of von Willebrand factor to prevent the survival of sizable multimers that cause a rise in thrombosis and platelet usage in the microvascular network (17). Our patient had severely reduced ADAMST13 activity due to an inhibitor, which was resolved in parallel with the clinical resolution of TMA symptoms, ruling out a genetic or persistent deficiency. The diagnosis of HUS *vs* TTP is typically determined based on the frequent renal complications of HUS and the common neurological symptoms of TTP; nonetheless, overlap between the two disorders is not uncommon (18). In this case, the help of the ADAMST13 information favored TTP, even though an eventual histological change in the renal involvement of her lupus could explain the severe renal damage the patient was facing. Obtaining a new renal histology would have been useful, but the procedure was not feasible due to the patient’s condition.

During pregnancy, the maternal-placental immune tolerance phenomenon produces an unresponsive state while maintaining immune system competence. This is accomplished through an increase in innate immunity and a reduction in adaptive immunity (19); when complement dysregulation is present, pregnancy acts as an additional trigger for complement-related disorders.

Initially, therapy was empiric, pending test results, and included plasmapheresis as essential to control thrombotic thrombocytopenic purpura. Upon obtention of the ADAMST13 study results showing a low, inhibitor-dependent, activity, the notion of the presence of severe lupus with autoimmune activity against ADAMST13 was solidified; consequently, the use of cyclophosphamide was validated as a viable solution. But biomarkers may not be available in all facilities and consequently, clinical scores have been formulated for evaluating hospitalized adults suspected of having TTP to determine if the early installation of plasma exchange is warranted while waiting for lab results (20); these clinical tools deserve a validation for their use during pregnancy (21).

It must be acknowledged that an efficient immunosuppressive induction of remission was impossible due to the pregnancy, which limited treatment to steroids and azathioprine, explaining why autoimmune activity persisted, included the production of the ADAMST13 inhibitor. This antibody, at the very least, played a role in causing the microangiopathic phenomenon. Antigen-antibody immune complexes of ADAMTS13 and anti-ADAMTS13 have the potential to activate the classical complement pathway (22).

One could speculate some relationship between the appearance of renal carcinoma and complement dysfunction, given the action of the latter on the immune cells, on the cancer cells and also action on angiogenesis (23), however, assuming the functional alteration was transient, it seems unlikely.

The oncogenic power of immunosuppressive therapy is another possible link between renal cancer and the patient’s initial disease. Although mycophenolate mofetil is generally considered safe for use in transplantation therapy (24) cyclophosphamide is an alkylating drug that can cause DNA changes leading to cytotoxicity and mutagenic effects that cannot be repaired if high doses are used for prolonged periods of time, especially in older patients (25); but this is not the case of our patient.

But the presence of neoplasia does not prohibit our patient from undergoing a future transplant. With the demonstration of an inhibitor of ADAMST13, the likelihood of TMA recurring in a subsequent transplant seems unlikely, given sufficient immune suppression. Currently, our center lacks genetic studies on alterations of the complement pathway; however, as the patient desires transplantation once free of tumor disease, we will make efforts to obtain a complete genetic study to assess the risk of recurrence and to evaluate the use of preventive therapy.

The mother’s life was saved through emergency treatment that was based on adequate clinical suspicion and confirmed by an accurate exploratory process. Regrettably, it failed to rescue the child and did not restore the mother’s kidney function. In a catastrophic scenario such as the one depicted for this patient, objective biomarkers (such as ADAMST13) are crucial for supporting complex therapies.

## Data availability statement

The datasets for this article are not publicly available due to concerns regarding participant/patient anonymity. Requests to access the datasets should be directed to the corresponding author.

## Ethics statement

Ethical review and approval was not required for the study on human participants in accordance with the local legislation and institutional requirements. Written informed consent was obtained from the patient for the publication of this case report.

## Author contributions

ML: Formal Analysis, Validation, Writing – original draft, Writing – review & editing, Data curation. GN: Data curation, Formal Analysis, Writing – review & editing, Conceptualization, Investigation, Visualization. JC: Data curation, Visualization, Writing – review & editing. LA: Conceptualization, Formal Analysis, Investigation, Visualization, Writing – review & editing, Methodology, Supervision, Validation, Writing – original draft.
